# Topics of Public Interest

**Published:** 1915-11

**Authors:** 


### TOPICS OF PUBLIC INTEREST.

**The Buffalo Ward.** Report by Dr. Feiss. Practically a year has elapsed since the American ambulance at Neuilly-sur-Seine opened its doors to receive wounded soldiers.

It goes without saying that most of the money which was collected to carry on the work of the ambulance came from America, and the city of Buffalo contributed enough to enable the hospital to start a little ward of its own, which has since been known as the "Buffalo Ward."

It is the pleasure of this committee to briefly describe the kind of cases treated, and the nature of the work that has been done in this ward, for the benefit of these friends of the Ambulance who have demonstrated their interest.

The Buffalo Ward has the distinction of being an Officers' ward. There is no difference between the treatment accorded to officers and common soldiers, but for obvious reasons and as is done in all war hospitals,officers are segregated together.

The ward is located in one of the best parts of the Ambulance both as regards sunlight and fresh air. It contains nine beds.

The cases are under the care of very efficient surgeons, while the nurse is one of the best known and kindest women in the Ambulance. She is helped in her work by an "auxiliary" nurse who is also very well trained in her duties.

Following are some of the cases which are now in the ward and which serve to illustrate the character of the work done:

2nd Lieut. D.... of the 23rd Infantry was wounded in the ankle last January during an attack north of Soissons. After a hasty dressing he lay in the trench all night and was brought from the trench to a dressing station the next morning under German fire. Then he was taken to the hospital at Soissons, which hospital had to be evacuated very soon after owing to the proximity of the enemy. He was sent to Vierzy where he was operated on and lated brought to this Ambulance.

Here, while in the Buffalo Ward he has been obliged to undergo a series of operations to remove fragments of dead bone. Now he is well on the road to recovery, and will soon be able to walk again although his stiff ankle may cause a slight limp.

Lying next to each other in this same ward are two captains, each with a compound fracture of the thigh.

One of these, Capt. Br.... belongs to a regiment of Zouaves. He was wounded one night while leading his company through some intricate manoeuvres to gain some German trenches. When he was hit, some of his Zouaves tried to carry him off to a place of greater safety, but the brave captain quickly ordered them away owing to the firing going on all about him. As the blood flowed freely from his wound he tried to stop it by tying his cravat about his leg, then he became unconscious and lost all notion of time. Later he was told that ten volunteers had come back after him and that after eight of these brave fellows had fallen, the survivors finally found him and brought him away from the field of battle back to a "Poste de Secours," where he was dressed. Finally he was he was operated on and later brought to this Ambulance.

This took place late in the fall and he has lain here in the Buffalo Ward ever since with a thigh fracture which does not completely unite. His general condition is splendid, and just at present it seems from the X-ray pictures that consolidation is going on well, so that it is hoped that before October comes he will be on his feet again.

His neighbor, Capt. Bu. . . ., belongs to the famous 1st Foreign legion. He was wounded last May while advancing with his company to take some German mitrailleuses by a flank attack. He sustained a fractured thigh. His orderly, a Swiss, was at his side at once, and dressed his wound. He suddenly noticed a body of the enemy approaching and proceeded to pick up the captain in order to carry him off on his back. But the load was too heavy for him and at last, at the suggestion of the wounded man himself, he finally succeeded in getting him out of danger by grasping him by the wrists and actually dragging him over the ground. He had to be dragged this way for a distance of four hundred metres.

Afterwards, he was brought by train to Paris, and arrived at the American Ambulance in rather bad condition.

Here, by means of an X-ray examination, the bullet which caused the damage was located, having become imbedded in one of the bone fragments. Later, an operation was done, the fracture carefully cleaned up and the bullet, a most precious souvenir, finally removed. This fracture has done very well, the bone being perfectly united in splendid position.

No patient has ever been so gracious and thankful as this one. He is always smiling, and has never been known to offer a complaint even at times when he was known to suffer greatly.

Lieut. Be. . . . belongs to a regiment of Chasseurs. He was wounded on April 26th at some place north of Ypres. His battalion had gained some trenches when the Germans sent a cloud of their asphyxiating gas at them, which obliged a hasty retreat. It was then that he was hit. The wound was of the face, and was quite fresh when he entered the Ambulance. It was an awful thing to gaze upon then. The bullet seemed to have entered through the neck and to have come out through the lower jaw of which only a small portion was left. Most of his tongue was shot away and his teeth and lower lip had completely disappeared. At the beginning he could neither talk nor eat. He had to be fed through the nose and has obtained all his nourishment this way for two months. His treatment has been in the hands of surgeons and dentists, who have succeeded in establishing a new jaw. His lips will have to be remodeled and he will be given a good set of teeth. At present he is talking quite distinctly, and can eat soft solids by mouth. He is a man of great patience and courage and is a great credit to the ward.

Lieut. La.... is another patient of great courage belonging to the 28th artillery. A bullet struck him while he was making observations from a parapet. Strange enough, the bullet entered his eye and passed out through his ear, fracturing the bone behind the ear. After having been dressed in a field hospital, he was brought here and kept under observation for a few days. Later he developed fever and had to be operated on in order to have pieces of dead bone removed. This was followed by several secondary hemorrhages which have weakened him a bit, but at the present time he is convalescent except for a paralysis of the facial nerve which is of course disfiguring. Some attempt will possibly be made later to restore the lost function of the nerve.

Finally, there is the case of Lieut. M.... of the 353d regiment of reserve, who was wounded during a surprise attack in which part of his regiment was surrounded. The bullet passed through his leg, and although the wound seemed of no great importance at first, the infection spread and his knee joint became involved. He was not treated in this Ambulance at the beginning, and at the time he was seen here he presented a leg so badly infected that amputation was considered. By careful treatment, however, the leg was saved, the patient undergoing several operations. Today he is so much improved that he goes about with crutches, and even expects to discard them soon.

Many other cases have been treated in this ward, but enough has been said to give an idea of the nature of some of the wounds and character of the men who have come under our care here.

All of these cases have shown the greatest patience and courage, and it has been a privilege rather than a task to have the care of them. If our kind friends in Buffalo could see for themselves what some of the results have been, we feel more than sure that they would quickly realize the importance of the work to which they have so graciously given their support.

---

**Vaccinations** (antivariolar) must be reported in N. Y. State, on special cards, with blanks for various details, including result of inspection. We understand that the cards may be obtained from the local health officer. At any rate, they are to be returned to him for forwarding to the state authorities. No one but a regularly licensed physician may now perform vaccination.

---

**Oral Examination** has been substituted by the Massachusetts Medical Society for written examination, previously required of its candidates. It is considered that graduation from a recognized college and written examinations for licensure is a sufficient protection against unworthy admissions.

---

**Deaths From Traffic in N. Y. City** for September numbered 57. Automobiles killed 43. Of the 57, 35 were children. For the state outside of N. Y. City, 63 were killed, and of these 57 by automobiles.

---

**The Population of Buffalo,** not including inmates of charitable and penal institutions, is 461,887, a gain of 38,172 in five years.

---

**The J. N. Adam Memorial Hospital** at Perrysburg, opened special wards for children on October 9. $5,000 was raised by school children in 1912, by the sale of Red Cross Christmas seals. This fund was subsequently doubled. The City of Buffalo also voted $120,000 for the erection of buildings needed. A special excursion was run for the opening and a memorial tablet was unveiled commemorating the work of the children.

---

**Clinical Lectures on Diseases of the Skin** will be given by Dr. Duncan Bulkley at the N. Y. Skin and Cancer Hospital, 2d Avenue and 19th Street, every Wednesday at 4.15.

---

**Hog Cholera** is reported at Pavilion, 50 deaths on one farm, and at Leroy and Churchville Dr. Nelson N. Leffler of Batavia is in charge and is inoculating exposed animals.

---

**Fumigation** following infectious diseases has been discontinued in Greater N. Y., statistics showing that no greater protection from subsequent infection has followed its use. Soap and water, burning of hopelessly infected bedding, steam renovation of other material, direct application of disinfectants, in bulk or by spraying, are better means of disinfection. Still, we feel, that under certain limitations, mainly as to penetrability and necessity of moisture, which are pretty well demonstrated scientifically, fumigation has a place in sanitation.

---

**Misbranded Medicines.** The U. S. Dept. of Agriculture has successfully concluded 50 suits under the Sherley amendment of the Pure Food and Drug Law. This amendment prohibits false and fraudulent claims as to the curative or therapeutic effects of a preparation. All but one of the medicines were of the ordinary patent medicine type. The exception was an "ethical proprietary," a simple, harmless preparation, of considerable value if properly and not too optimistically used and one as to the composition of which any physician could easily inform himself. Misbranding, in such a case, must have consisted in too highly colored statements of therapeutic value.

---

In the Construction of **Arrow Rock Dam,** in Idaho, about 20,000 men were employed, the maximum at any one time being 1500. There was no death from infectious disease in four years and less than a dozen deaths from accident. These facts speak for the efficiency of the medical and surgical and sanitary service. Analogously, the dam was completed in 4 instead of 5 years, and at a total expense of 5 instead of 7½ millions, as estimated. As in the case of the Panama Canal, honesty and efficiency are likely to show themselves at the same level in all departments of an undertaking.

---

**Proposed College Merger.** The Hahnemann Medical College of San Francisco has offered to convey its property to the University of California, if two chairs of Homoeopathic Materia Medica and Therapeutics are maintained by the latter, as facultative, not obligate courses of study. This seems to be a fair offer and, by the way, why should not every college maintain such chairs? This does not imply a personal bias but we believe that a full hearing of "the other side," always tends toward ultimate truth.

---

**The Housewives' League.** Mrs. Arthur S. Hurrell, President of the Buffalo organization calls our attention to the relation of the Health Dept. to domestic economy and sanitation. Even the Educational system has scarcely so close a relation to the home, since the education of children is dependent on their health, not to mention that many homes contain no children of school age. Modern housewives realize that they are engaged in a serious business. The kitchen factory should have the same standards of efficiency, supported by public departments, as any other factory. The raw materials bought for elaboration in the former require the same control as to fairness of price and testing as to quality, as in the case of the latter. Indeed, the requirement of the former is even more insistent since not only material wealth but health depend upon it. The majority of food materials purchased inevitably contain 10-50% of commercial waste, that is to say of inedible or innutritious matter which cannot practically be separated from the raw material till it is prepared in the kitchen or even till it is served. In addition, even the edible portion may, in the case of coarse vegetables, contain less than 5% of actually nutritious substances and this percentage seldom rises above 50, except in the case of artificially prepared foods such as sugar, flour, starch, butter and oils. Thus, quite apart from direct sanitary considerations, the addition of a further element of waste by putrefaction, fermentation, gross contamination with dirt and flies, represents in the aggregate, the purchase of tons of raw material that must be discarded.

The Housewives' League, therefore, calls attention to the need of further legislation and provision for inspectors, to maintain the markets of the city in a state of cleanliness, particularly as to the exclusion of flies, removal of garbage, and provision for the personal cleanliness of those selling their wares.

---

**Automobiles.** New York State, with a population of 9,700,000, has 222,024 automobiles this year, a ratio of 1 : 43 plus, of the population. This means one to about 8 families, except that many are commercial, although the frequency with which commercial and dealers' licenses are seen in the country, indicates that the substraction on this account is not as great as might be expected.

